# RNA‐seq bulked segregant analysis combined with KASP genotyping rapidly identified *PmCH7087* as responsible for powdery mildew resistance in wheat

**DOI:** 10.1002/tpg2.20120

**Published:** 2021-07-26

**Authors:** Haixian Zhan, Yingli Wang, Dan Zhang, Chenhui Du, Xiaojun Zhang, Xiaoli Liu, Guangyuan Wang, Shuosheng Zhang

**Affiliations:** ^1^ Institute of Pharmaceutical & Food Engineering Shanxi Univ. of Chinese Medicine Jingzhong 030619 China; ^2^ College of Agriculture Shanxi Agricultural Univ. Taiyuan 030032 China

## Abstract

Powdery mildew causes considerable yield losses in common wheat (*Triticum aestivum* L.) production. Mapping and cloning powdery mildew‐resistant quantitative trait loci can benefit stable yield production by facilitating the breeding of resistant varieties. In this study, we used the powdery mildew resistance introgression line ‘CH7087’ (harboring the resistance gene *PmCH7087*) and developed a large F_2_ population and a corresponding F_2:3_ segregation population containing 2,000 family lines for molecular mapping of *PmCH7087*. Genetic analysis demonstrated that the resistance phenotype was controlled by a single dominant gene. According to the performance exhibited by the F_2:3_ lines, 50 resistant lines and 50 susceptible lines without phenotype segregation were chosen for pooling and bulked segregant RNA sequencing (BSR‐Seq) analysis. A region spanning 42.77 Mb was identified, and genotyping of an additional 183 F_2:3_ lines with extreme phenotypes using 20 kompetitive allele‐specific polymerase chain reaction (KASP) markers in the BSR‐Seq mapping regions confirmed this region and narrowed it to 9.68 Mb, in which 45 genes were identified and annotated. Five of these transcripts harbored nonsynonymous single nucleotide polymorphisms between the two parents, with the transcripts of *TraesCS2B01G302800* being involved in signal transduction. Furthermore, *TraesCS2B01G302800.2* was annotated as the closest homologue of serine/threonine‐protein kinase PBS1, a typical participant in the plant disease immune response, indicating that *TraesCS2B01G302800* was the candidate gene of *PmCH7087*. Our results may facilitate future research attempting to improve powdery mildew resistance in wheat and to identify candidate genes for further verification and gene cloning.

## Core Ideas

- A span of 42.77 Mb and 385 genes on chromosome 2B were annotated.
- Based on the KASP genotypes, seven SNP markers were associated with *PmCH7087*.
- *TraesCS2B01G302800* was the most likely candidate gene for *PmCH7087*.

**Abbreviations**
BSR‐Seq: bulked segregant RNA sequencing
DEG: differentially expressed gene
IT: infection type
KASP: kompetitive allele‐specific polymerase chain reaction
MAS: marker‐assisted selection
*Pm*
: Powdery mildew resistance genes
QTL: quantitative trait locus
R01: resistant parent CH7087 RNA sample group
R02: homozygous resistant bulk RNA sample group
S01: susceptible parent Taichung 29 RNA sample group
S02: homozygous susceptible bulk RNA sample group
SNP: single nucleotide polymorphism

## 1 INTRODUCTION

Common wheat (*Triticum aestivum* L) is one of the major crops providing food energy to humans, and its stable reproduction is threatened by various diseases (Wellings, 2011). Powdery mildew, one of the most devastating diseases affecting wheat, is caused by *Blumeria graminis* f. sp. *tritici* and can cause not only dramatic yield losses but also decreased wheat grain quality in common wheat worldwide (Singh et al., 2016). Identifying and utilizing new genes to breed cultivars with improved disease resistance have long been a crucial and effective strategy formed for overcoming these biotic stresses (Nelson et al., 2018). Marker‐assisted selection can accelerate these breeding programs (Anderson, 2009; Yaniv et al., 2015). Increasing numbers of powdery mildew resistance genes and quantitative trait loci (QTLs) have been reported in the gene pool of elite wheat varieties and its wild relatives (Li, Dong, Ma, et al., 2020; Li, Dong, Li, et al., 2020; Lu et al., 2020; Qie et al., 2019; Xie et al., 2020). However, major resistance genes generally are overcome rapidly when they are applied over larger areas and for longer periods of time because of changes in virulence and the appearance of novel races in the pathogen population. Therefore, it is necessary to investigate novel resistance gene pools and use long‐lasting disease resistance genes to breed resistant wheat cultivars.

Quantitative trait loci (QTLs) responsible for resistance to powdery mildew were first characterized decades ago (Keller et al., 1999). A review reported that over 102 formally designated powdery mildew (*Pm*) resistance genes have been genetically identified in wheat and its wild relatives. These genes, which tend to be located in the A or B subgenome (Guo et al., 2017), include *Pm16* (Chen et al., 2005), *Pm26* (Rong et al., 2000), *Pm30* (Liu et al., 2002), *Pm36* (Blanco et al., 2008), *Pm41* (Wang et al., 2014), *Pm42* (Hua et al., 2009), *Pm34* (Miranda et al., 2006), *Pm35* (Miranda et al., 2007), *Pm58* (Wiersma et al., 2017) from *Aegilops tauschii* Coss, and *Pm67* (Zhang et al., 2020). More recently, many *Pm* genes were finely mapped and the functions of some were determined, including *Pm1a* (Hewitt et al., 2020), *Pm2* (Sánchez‐Martín et al., 2016), *Pm3*, *Pm8* (Srichumpa et al., 2005), *Pm4b* (Wu et al, 2018), *Pm5* (Qie et al., 2019), *Pm5e* (Xie et al., 2020), *Pm64* (Zhang et al., 2019), and *Pm66* (Li, Dong, Ma, et al., 2020). In a high‐density genetic map, *Pm52* was finely mapped in a 0.21‐cM genetic interval corresponding to a 5.6‐Mb genomic region on chromosome arm 2BL (Wu et al., 2019). A wide spectrum of resistance to the *B. graminis* f. sp. *tritici* allele *Pm63* was located in an interval of approximately 13.1 Mb on the long arm of chromosome 2B by traditional linkage mapping (Tan, Li, et al., 2019). Notably, several *Pm* genes were cloned and well characterized functionally. *Pm3b*, conferring race‐specific resistance, confers coiled‐coil nucleotide binding site–leucine‐rich repeat (Yahiaoui et al., 2004). Sánchez‐Martín et al. reported two cloned genes *Pm2 and Pm4*, the latter of which encoded a putative chimeric protein of a serine/threonine kinase and multiple C2 domains and transmembrane regions (Sánchez‐Martín et al., 2016; Sánchez‐Martín et al., 2021). *Pm21*, encoding a typical coiled‐coil nucleotide binding site–leucine‐rich repeat protein, confers broad‐spectrum resistance to powdery mildew disease in wheat (He et al., 2018). Through homology‐based cloning, the *Pm8* and *Pm17* were isolated on the 1RS introgression in wheat cultivar ‘Amigo’ (Singh et al., 2018). Through a map‐based strategy, *Pm24* was cloned; this gene encodes the WTK3 protein with putative kinase‐pseudokinase domains and is a broad‐spectrum powdery mildew resistance gene (Lu et al., 2020). *Pm41*, located on chromosome 3BL, encodes the CNL protein and confers powdery mildew resistance (Li, Dong, Li, et al., 2020; Wang et al., 2014).

**Core Ideas**

- A span of 42.77 Mb and 385 genes on chromosome 2B were annotated.
- Based on the KASP genotypes, seven SNP markers were associated with *PmCH7087*.
- *TraesCS2B01G302800* was the most likely candidate gene for *PmCH7087*.

Bulked segregant RNA sequencing (BSR‐Seq) is a modified bulked segregant analysis for mapping genes and was first developed and applied in maize (Zea mays L.), a species with a relatively larger genome size (Liu et al., 2012), and subsequently introduced to wheat as described above. Segregant RNA sequencing used RNA‐seq data to call single nucleotide polymorphisms (SNPs), which balances the sequencing cost and molecular marker density for species with a large genome. In addition, expression information, especially regarding differentially expressed genes (DEGs), can provide the necessary information for gene screening, especially for adverse stress‐related traits, such as disease resistance. The mapping resolution of BSR‐Seq has also been determined to be satisfactory (Liu et al., 2012). Recently, many genes from different crops that are responsible for disease resistance, abiotic stress, and development have been reported. *Pm4b* was reported to be located on chromosome 2AL by BSR‐Seq and further mapped in a 3.0‐cM genetic interval spanning 6.7 Mb (Wu et al., 2018). A two‐step BSR‐Seq approach identified *Pm5e*, which encodes a leucine‐rich‐repeat‐containing protein, and a rare nonsynonymous single nucleotide variant in which the *C*‐terminal leucine‐rich repeat domain was determined to confer the resistance to powdery mildew (Xie et al., 2020). *Yr15*, a wheat yellow rust disease resistance gene, was mapped to a 0.77‐cM interval by implementing BSR‐Seq and its corresponding SNP markers were developed (Ramirez‐Gonzalez et al., 2015). Through a combination of BSR‐Seq and linkage analysis, *Pm61* was located in a 0.71‐cM genetic interval in common wheat (Hu et al., 2019). In the Chinese cabbage (*Brassica rapa* L. ssp. *pekinesnis*) DH line ‘FT’, the male sterile gene *ftms* was mapped onto chromosome A05 via BSR‐Seq analysis, and the region was narrowed to approximately 1.7 Mb via linkage analysis (Tan, Liu, et al., 2019). To isolate waterlogging‐tolerance genes in maize, however, a set of 10 waterlogging sensitive and eight waterlogging‐tolerant inbred lines were adopted for BSR‐Seq, and six alleles were identified by integrating the DEG data and genomic mapping positions (Du et al., 2017).

Tall wheatgrass [*Thinopyrum ponticum* (Podp.) Z.‐W. Liu & R.‐C. Wang (syn. *Agropyron elongatum* [Host] Beauv., *Lophopyrum ponticum* [Podp.] A. Löve, and *Elytrigia elongata* [Host] Nevski)] has been proven to be a valuable source of disease resistance in wheat (Li & Wang, 2009). The resistance genes can be introgressed into wheat cultivars by wheat–*T. ponticum* distant hybridization. The introgression CH7087 has demonstrated high resistance to powdery mildew in wheat. In the present study, a large F_2_ population was developed by crossing CH7087 with ‘Taichung 29’, a powdery mildew‐susceptible cultivar. A subsequent F_2:3_ family population with 2,000 lines was also developed. After phenotyping, BSR‐Seq of bulked pools was used to identify SNPs associated with powdery mildew resistance. Subsequently, we developed kompetitive allele‐specific polymerase chain reaction (KASP) markers to genotype the extreme F_2:3_ lines to further confirm and narrow down our mapping region. Nonsynonymous SNP annotations, gene enrichment analyses, and DEGs were combined to screen thecandidate gene. The markers linked to *PmCH7087* may be useful for marker‐assisted selection in wheat breeding programs and map‐based cloning of *PmCH7087*.

## 2 MATERIALS AND METHODS

### 2.1 Materials

The powdery mildew‐resistant accession ‘R431’, derived from *T. ponticum* and the common wheat cultivar Taichung 29, was provided by Shanxi Agricultural University, Taiyuan. CH7087 is an introgression line with high powdery mildew resistance, whereas Taichung 29 is highly susceptible to powdery mildew disease. To develop high‐resolution molecular mapping of the resistant QTLs underlying CH7087, an F_2_ segregation population with 2,372 individuals was developed by crossing CH7087 with Taichung 29 (equivalent to a backcross). The corresponding F_2:3_ families were also developed for powdery mildew resistance phenotyping, and the performance of every F_2:3_ family individual was used to evaluate the corresponding F_2_ resistance.

### 2.2 Powdery mildew resistance testing

Powdery mildew resistance was evaluated at the seedling stage. The CH7087, Taichung 29, ‘SY95‐71’, F_1_ hybrid and F_2:3_ families were grown in a greenhouse with a light time of 18 h and a dark time of 6 h at 26 ℃. The resistance of CH7087, Taichung 29, SY95‐71, and part of the F_1_ hybrid populations were evaluated in 2016. The parents and F_2_ populations were evaluated in 2017. Four seeds were planted discretely in a 4‐ by 4‐ by 4‐cm plug tray. Twenty seeds from all three parents (CH7087, Taichung 29 and SY95‐71) and the F_1_ hybrid were sown into mixed peat with vermiculite (2:1). Approximately 15 seeds were sown for every F_2:3_ family line. At the one‐leaf stage, the spores of *B*. *graminis* f. sp. *tritici* isolate E09 were inoculated artificially and water was sprayed to maintain wetness. To ensure successful infection, we performed inoculations two times in the morning on two continuous days. The wheat cultivar Mianyang 11 was used as a susceptible control. After the susceptible control was heavily infected (approximately 7 d after inoculation), the infection types (ITs) were evaluated on a 0 to 4 infection type scale according to a previously described method (He et al., 2009). The appearance of F_2:3_ lines was recorded three times to ensure the reliability of the disease resistance measurements. For seed reproduction of the F_2_ and F_2:3_ families, sowing, watering, and fertilizer management were the same as in field production. In order to understand the response to different strains, the performance of CH7087 were evaluated as well in the light incubator with different *B. graminis* f. sp. *tritici* strain (E20, E21, and E26). The evaluation conditions and methods were the same as above.

### 2.3 RNA library construction and Illumina sequencing

Individuals of one family line with a consistent phenotype in the F_2:3_ population were chosen to guarantee accurate IT pooling. Fifty homozygous resistant lines (IT = 0) and 50 homozygous susceptible lines (IT = 4) of the F_2:3_ families were screened. Leaves of 10 randomly selected F_2:3_ lines after inoculation for 7 d were collected and mixed equally to extract the total RNA, which was treated as the corresponding genotypes of F_2_. The leaves of the parents CH7087 and Taichung 29 after inoculation for 7 d were also sampled. Total RNA was extracted by the total RNA extractor (Sangon Biotech). Equivalent RNA of every single F_2:3_ line was pooled to construct resistant and susceptible bulks. Finally, RNA samples were divided into four groups: the resistant parent CH7087 (R01), the susceptible parent Taichung 29 (S01), homozygous resistant bulks (R02), and homozygous susceptible bulks (S02) for subsequent sequencing library construction. After measurement of the concentration with a Nanodrop Drop 2000 spectrophotometer (Thermo Scientific) and quality control with an Agilent Bioanalzer 2100 (Agilent Technologies), cDNA libraries were generated. All the libraries were loaded into one lane of a flow cell and sequenced by an Illumina X‐TEN platform with the PE150 model according to the manufacturer's instructions.

### 2.4 Differentially expressed gene analysis

Clean data of the four groups were obtained by filtering sequencing reads with more than 50% of bases having a Q value of ≤10 or an ambiguous sequence content exceeding 10%. Next, the remaining reads were aligned to the wheat genome sequence (https://urgi.versailles.inra.fr/download/iwgsc/IWGSC_RefSeq_Assemblies/v1.0/, accessed 28 May 2021) by TopHat2 software with the default settings (Kim et al., 2013). The components of Cuffquant and Cuffnorm were used to determine the quantitative expression as fragments per kilobase of transcript per million fragments mapped (Trapnell et al., 2010). DESeq (Anders & Huber, 2010) was used for calling DEGs at the level of fold change ≥2 and a false discovery rate of  <0.01. Further detailed information for typical RNA‐seq analysis was obtained according to previously described methods with slightly modified points (Xue et al., 2017).

### 2.5 Single nucleotide polymorphism calling and molecular mapping

After TopHat2 mapping was performed, the GATK HaplotypeCaller module was used for SNP calling in parallel (McKenna et al., 2020) and SnpEff was used for SNP annotation (Cingolani et al., 2012). Single nucleotide polymorphisms with a sequencing depth of <4 in all four groups were filtered. The SNP‐index algorithm (Takagi et al., 2013) was used to estimate the association between high‐quality SNPs and powdery mildew. SNPNUM was used to fit the SNP‐index values (Li et al., 2009). Powdery mildew resistance‐associated loci were identified when the fitted values of the SNP‐index were above the threshold at the 99% confidence interval (Zhang et al., 2018). A circos graph containing chromosomes, genes, and SNP density was generated by CIRCOS 0.66 software (http://circos.ca/, accessed 28 May 2021).

### 2.6 Bulked segregant RNA sequencing mapping confirmation and candidate gene screening

Bulked segregant RNA sequencing analysis resulted in an region associated with powdery mildew resistance. To confirm and narrow the region, we used KASP markers in an additional panel of individuals with extreme phenotypes from the F_2:3_ population. We selected 30 SNPs evenly distributed throughout the region and converted them into KASP markers for population genotyping. The primer information is shown in Supplemental Table S1. In total, 183 BSR individual‐free F_2:3_ families, including 136 resistant families and 47 susceptible families, were genotyped by KASP markers, and a further *t*‐test was used to generate new regions harboring significantly different SNPs. Moreover, nonsynonymous SNPs and DEGs between the two parents (R01 vs. S01) and between the resistant and susceptible bulks (R02 vs. S02) were identified in the new region. Gene Ontology (Ashburner et al., 2000), Kyoto Encyclopedia of Genes and Genomes (Kanehisa & Goto, 2000), eggNOG (Huerta‐Cepas et al., 2016), and NR (Deng et al., 2006) database enrichment analyses were conducted to help identify potential candidate genes.

## 3 RESULTS

### 3.1 Genetic analyses of the resistant phenotype

The performance of CH7087, Taichung 29, and the F_1_ hybrid and F_2:3_ families inoculated with *B. graminis* f. sp. *tritici* isolate E09 was evaluated. To simplify phenotyping, plants with scores between 0 and 2 points were regarded as resistant; the rest were marked as susceptible. Typical performance scores of 0 to 4 are shown in Supplemental Figure S1. As shown in Table 1, CH7087 and the F_1_ population were highly resistant (IT = 0), whereas Taichung 29 and SY95‐71 were highly susceptible (IT = 4). The data from F_2:3_ families segregated as 533 homozygous resistant: 1,102 segregating: 520 homozygous susceptible (χ^2^1:2:1 = 1.271, *P*_df2_ = .530) (Table 1). The segregation ratio was in keeping with previous results that powdery mildew resistance was indicative of dominant inheritance in CH7087. The resistant gene was temporarily designated as *PmCH7087*.

**TABLE 1:** The segregation patterns of F_1_ plants, F_2_:_3_ families, and their parents inoculated with powdery mildew

Materials	Generation	No. of plants or lines			Expected ratio	χ^2a^	*P*
		Resistance	Segregation	Susceptibility			
CH7087	P_1_	20	0	0	–	–	–
Taichung 29	P_2_	0	0	19	–	–	–
SY95‐71	P_3_	–	–	20	–	–	–
SY95‐71 × CH7087	F_1_	25	0	0	–	–	–
	F_2_	123		47	3:1	0.635	0.426
CH7087 × Taichung 29	F_1_	25	0	0	–	–	–
	F_2_	1,798		574	3:1	0.812	0.368
	F_2:3_	533	1,102	520	1 : 2 :1	1.271	0.530

*Note*:Values were significant at *P* =.05, and at χ2 = 3.83 and 5.99 for one and two degrees of freedome, respectively.

### 3.2 Bulked segregant RNA sequencing analyses

The BSR‐Seq strategy was used to map the resistance gene *PmCH7087*. RNA sequencing produced 12.74, 13.24, 40.54, and 40.02 Gb of clean data in R01, S01, R02, and S02, respectively. The percentage of the Q30 value (the Q30 valued s is equivalent to the probability of an incorrect base call 1 in 1,000 times) ranged from 93.04 to 93.61%, and the GC content of each sample ranged between 56.55 and 58.08%. In addition, the mapped ratio was between 84.27% and 86.67% for the four samples (Table 2). These results indicated that a relatively reliable dataset was generated for follow‐up analysis. The clean sequencing data were uploaded to the Sequence Read Archive database (accessions: SRR10019739, SRR10019740, SRR10019741, and SRR10019742).

**TABLE 2:** RNA sequencing data overview

Sample ID	Sample description	Clean reads^a^	Clean bases	GC content	Q30	Mapped reads	Mapped ratio
		*n*	Gb	%		*n*	%
R01	CH7087	85,265,454	12.74	58.08	93.54	73,445,934	86.14
S01	Taichuang 29	88,684,702	13.24	57.74	93.04	76,866,993	86.67
R02	Resistant bulks	271,366,656	40.54	57.16	93.24	234,930,724	86.57
S02	Susceptible bulks	274,623,910	40.02	56.55	93.61	231,427,664	84.27

^a^ Clean reads: the number of pair‐end reads in the clean data; Clean bases: the total number of bases in the clean data; GC content: the GC content of the clean data; Q30: The percentage of bases for which the quality value of the clean data was ≥30; Mapped reads: the number of clean reads mapped on the reference genome; Mapped ratio: the percentage of mapped reads in the clean reads.

**FIGURE 1:**
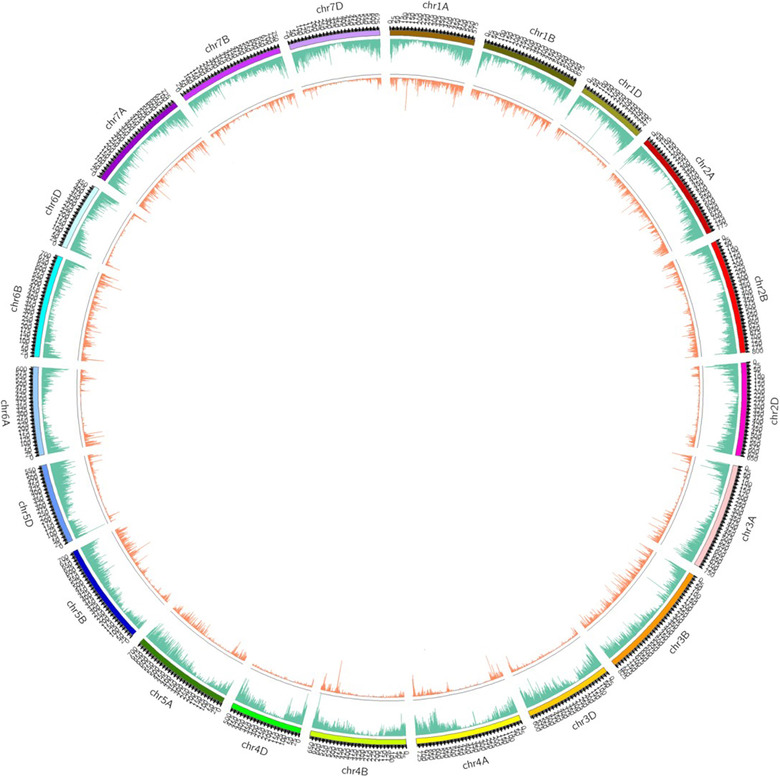
Circos plot of genome‐wide genes and single nucleotide polymorphisms (SNPs) distributions. The outer circle indicates chromosomes; the middle circle indicates gene distribution, and the inner circle indicates the SNP density distribution

We further identified 64,999 expressed genes from the four sequencing libraries and 64,709 SNPs were called between the parent CH7087 and Taichung 29 in total (Figure 1, Table 3). After filtering, we obtained 10,187 high‐quality SNPs. The SNPs were roughly enriched on the long arms and short arms of the chromosome (Figure 1). Table 3 shows the information of these SNPs. The average SNP density was 0.68 SNPs per Mb, with the highest density being in chromosome 3B (1.10 SNPs per Mb) and the lowest density being in chromosome 4A (0.47 SNPs per Mb). The SNP sequencing depth of the parents (R01 and S01) fluctuated between 7.75× to 9.49×, and the two pools ranged from 20.44× to 22.94×; 2,608 of the 10,187 SNPs were nonsynonymous (Table 3).

**TABLE 3:** Single nucleotide polymorphism (SNP) characteristics for bulked segregant RNA sequencing mapping

Chromosome	SNP number	SNP density	Average depth of R01^a^	Average depth of S01	Average depth of R02	Average depth of S02	Nonsynonymous SNP number
		SNPs per Mb					
1A	583	0.98	8.02	9.42	22.74	21.64	146
1B	552	0.80	8.18	8.90	21.58	21.42	95
1D	322	0.65	8.27	8.74	21.38	22.41	65
2A	632	0.81	8.37	8.97	22.74	21.77	180
2B	637	0.79	8.02	8.97	21.30	21.51	187
2D	502	0.77	8.44	8.81	23.14	22.09	123
3A	525	0.70	8.15	8.79	22.39	21.76	140
3B	917	1.10	8.03	9.17	21.51	20.90	180
3D	322	0.52	8.45	9.12	21.74	20.44	100
4A	353	0.47	8.85	9.14	22.69	21.83	99
4B	466	0.69	8.24	8.61	22.33	21.87	120
4D	253	0.50	8.61	8.83	21.74	21.20	76
5A	496	0.70	8.55	8.92	22.20	22.00	137
5B	598	0.84	8.04	8.92	22.90	21.19	198
5D	381	0.67	8.34	8.99	22.93	21.38	115
6A	373	0.60	7.75	9.09	21.15	21.26	95
6B	487	0.68	8.11	8.94	22.11	21.08	102
6D	252	0.53	8.00	8.56	23.30	20.90	76
7A	446	0.61	8.13	9.11	22.50	21.87	123
7B	543	0.72	8.13	9.49	21.49	21.10	124
7D	455	0.71	8.27	8.65	22.94	22.33	113
Unanchored	92	0.19	8.99	9.17	20.57	2.27	14
Total	10187	0.68	8.27	8.97	22.15	20.65	2608

^a^ R01, resistant parent CH7087; S01, susceptible parent Taichung 29; R02, homozygous resistant bulks; S02, homozygous susceptible bulks.

**FIGURE 2:**
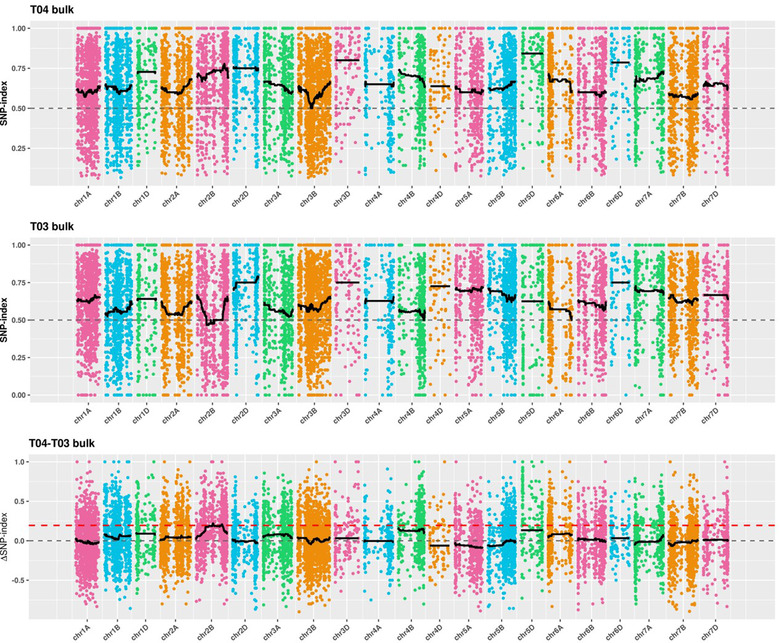
Segregant RNA sequencing (BSR‐Seq) mapping of *PmCH7087*. The *x*‐axis represents the position of 21 wheat chromosomes and the *y*‐axis represents the single nucleotide polymorphism (SNP)‐index/△SNP‐index. The red dashed line indicates the association threshold and the black line indicates the Loess‐fitted values

To map *PmCH7087*, individuals with extreme phenotypes were pooled for BSR‐Seq analysis. Two regions on chromosome 2B (425,795,642–453,022,545; 666,652,840–682,194,230) were estimated to be associated with powdery mildew disease (Figure 2). These regions spanned 42.77 Mb, and 385 genes were annotated in the two regions by the reference genome (Chinese Spring reference genome V1 version), including 25 genes harboring nonsynonymous SNPs between the two parents.

### 3.3 Confirmation and further mapping of *PmCH7087*

Since many genes were annotated in the BSR‐Seq mapping region, 30 KASP markers spanning from 425,795,642 to 682,194,230 bp on chromosome 2B were used to genotype additional F_2_ individuals for confirmation and further mapping of *PmCH7087*. Out of 30 SNPs, 20 were genotyped successfully in the two parents, which was in keeing with the SNPs called by RNA sequencing, and were further used for genotyping.

**FIGURE 3:**
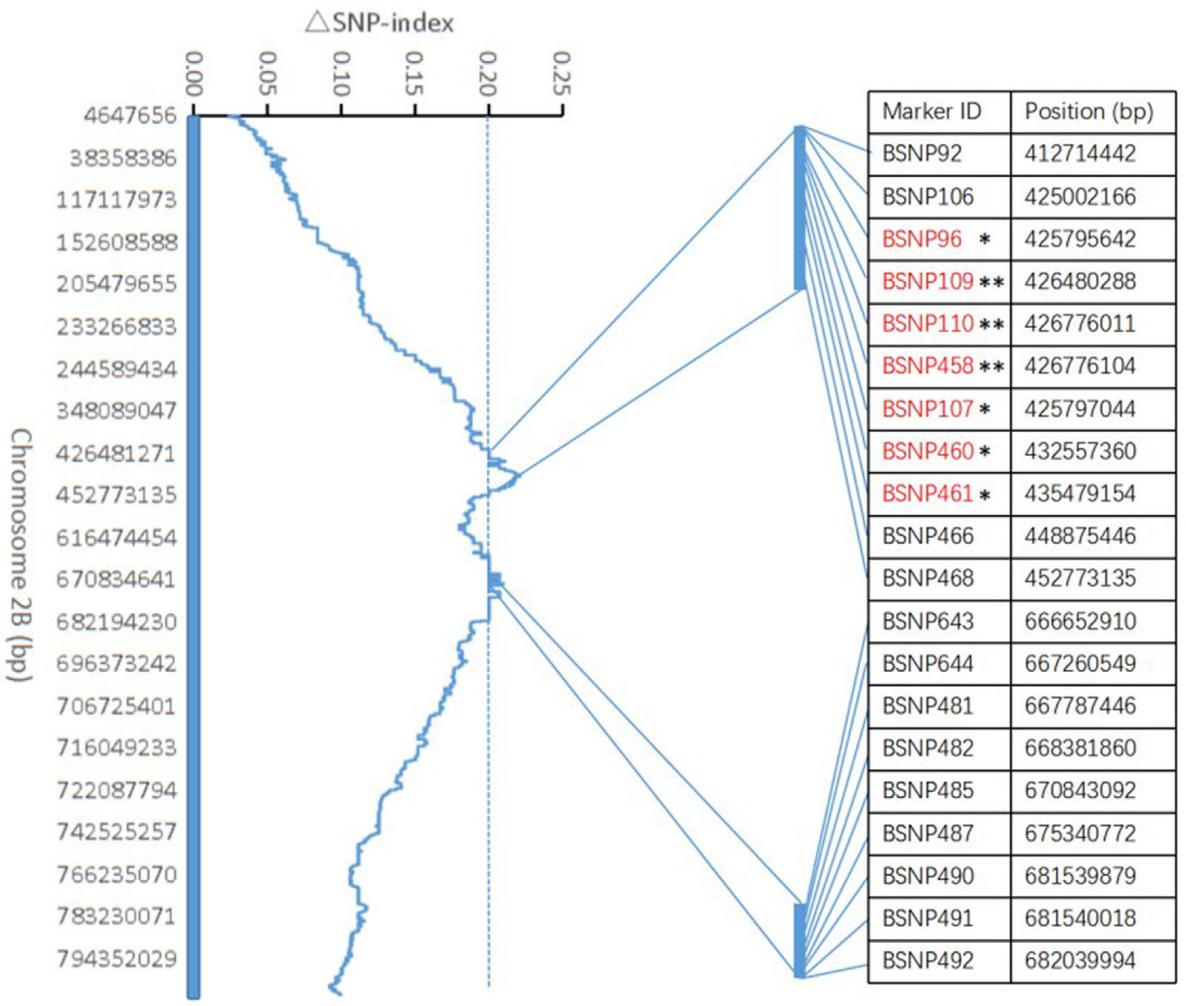
Confirmation and further mapping of *PmCH7087*. The left‐hand plot represents the association results of segregant RNA sequencing BSR‐Seq on chromosome 2B of wheat. The red ID numbers indicate significant (*, .01 < *P* < .05) or extremely significant (**, *P* < .01) linkages of single nucleotide polymorphisms with *PmCH7087*

On the basis of the the KASP genotypes between the contrasting phenotyping individuals, a *t*‐test demonstrated that seven continuous SNP markers (from BSNP96 to BSNP461) showed significant differences (Figure 3), indicating that these SNP markers were associated with *PmCH7087*. This region was between 425,795,642 and 435,479,154, a smaller region of 9.68 Mb, in which 45 transcripts were annotated; five of these transcripts harbored nonsynonymous SNPs between the two parents (Supplemental Table S2). Furthermore, the expression status of this 9.68 Mb region in the Chinese Spring reference genome was investigated. The DEG analysis demonstrated that no gene was upregulated or downregulated between R01 and S01 and between R02 and S02. Gene enrichment analyses showed that *TraesCS2B01G302800.2* and *TraesCS2B01G302800.1* were annotated in signal transduction mechanisms by the eggNOG database. Moreover, *TraesCS2B01G302800.2* was annotated as a serine/threonine‐protein kinase PBS1 by the NR database (Supplemental Table S2). These results indicated that *TraesCS2B01G302800* was the candidate gene regarding *PmCH7087*, but this possibility warrants further confirmation and verification of the deep molecular functional.

### 3.4 Comparison of *PmCH7087* and other powdery mildew resistance genes on chromosome 2BL

The powdery mildew resistance genes *Pm6* (Qin et al., 2011), *Pm33* (Zhu et al., 2005), *MlZec1* (Mohler et al., 2005), *Pm63* (Tan, Li, et al., 2019a), *Pm52* (*MlLX99*) (Wu et al., 2019), and *Pm64* (Zhang et al., 2019) were previously mapped on the long arm of chromosome 2B. The six genes were derived from *Triticum timopheevii* (Zhuk.) Zhuk., *Triticum carthlicum* Nevski, *Triticum dicoccoides* (Körn. ex Asch. & Graebn.) Schweinf., wild emmer [*Triticum dicoccon* (Schrank) Schübl.], and common wheat, respectively. Unlike these genes, *PmCH7087* was introgressed from *T. ponticum. Pm6* was susceptible to all strains except for strain E05. *Pm33* was susceptible only to strain E15 but was resistant to strains E03, E09, E20, and E26. *Pm51* provided resistance to strains E09, E20, E21, and E26. *Pm52* was resistant to E03, E09, E20, and E21, whereas *PmCH7087* was susceptible to E20 (Table 4). When we compared the powdery mildew reaction patterns of these genes, *PmCH7087* differed from *Pm6*, *Pm33*, *MlZec1*, *Pm63*, *Pm52*, and *Pm64* in its reactions.

**TABLE 4:** Resistance and physical location of eight genes

	*Bgt* ^a^ isolate								
Gene	E03	E15	E09	E20	E21	E26	Markers	Physical map	Reference
								Mb	
*Pm6*	S	R	S	S	S	S	*CIT02g‐20*	699.2	Qin et al., 2011
*Pm33*	R	–	R	R	S	R	X*wmc317*	Unmapped	Zhu et al., 2005
*MlZec1*	–	–	R	–	–	–	X*wmc356*	Unmapped	Mohler et al., 2005
*Pm51*	–	–	R	R	R	R	X*wmc332*	706.5	Zhan et al., 2014
*Pm52*	R	–	R	R	R	–	X*icssl326*	581	Wu et al., 2019
*Pm63*	–	–	–	–	–	–	X*stars419*	710.3	Tan, Li, et al., 2019
*Pm64*	–	–	R	–	–	–	*WGGBH1364*	695.4	Zhang et al., 2019
*PmCH70877*	–	–	R	S	R	R	*BSNP96*	425.8	–

^a^ *B. graminis* f. sp. *tritici*.

## 4 DISCUSSION

### 4.1 *PmCH7087* is a single dominant gene for wheat powdery mildew resistance

In the present study, we investigated the genetic characteristics of *PmCH7087* and found that all F_1_ plants were disease‐resistant, and a typical Mendelian 3:1 segregation ratio of the F_2_ population demonstrated that the resistance of powdery mildew was controlled by a single dominant gene (Table 1). More importantly, *PmCH7087* was mapped to chromosome 2B by BSR‐Seq (Figure 2), which was different from the locus reported earlier (Bin 2BL: 0.89–1.00) (Zhan et al., 2014), indicating different underlying powdery mildew resistance mechanisms in *T. ponticum*.

Several previously identified QTLs have been mapped onto the chromosome 2B, such as *Pm6*, *Pm33*, *Pm51* (Zhan et al., 2014), *Pm52*, *Pm63*, *Pm64*, *MlZec1*, *PmJM22* (Yin et al., 2009), and *MlAB10* (Maxwell et al., 2014). To investigate the relationship between *PmCH7087* and these genes, we mapped the corresponding linked markers of these QTLs onto the Chinese Spring reference genome V1 (Supplemental Table S3). No shared region was observed between *PmCH7087* and these QTLs except for *Pm33*, *Pm52*, and *MlZec1*, which had been mapped previously. In addition, different disease resistance patterns exist between *PmCH7087* and other gene series. The source, resistance phenotype, and position of the resistance genes indicate that *PmCH7087* may represent a novel resistance gene resource.

### 4.2 *TraesCS2B01G302800* is a putative candidate gene for *PmCH7087*

Since 385 genes were annotated in the primary mapping region, we further developed 20 SNP‐based KASP markers that were evenly distributed throughout the two regions, and we genotyped 183 individuals (136 resistant individuals and 47 susceptible individuals) of the F_2_ population with extreme performance. The *t*‐test resulted in seven KASP markers associated with disease resistance, which covered 9.68 Mb (425,795,642–435,479,154 bp). No associated SNP was detected in the region from 666,652,840 to 682,194,230 bp identified by BSR‐Seq, suggesting that these two regions were false positives. The confirmed region (425,795,642–435,479,154 bp) was 75 Mb from the centromere region of chromosome 2B (344.4–351.3 Mb) (The International Wheat Genome Sequencing Consortium et al., 2018; Walkowiak et al., 2020), indicating that limited recombination events occurred in the region beyond the end of the chromosome.

The protein kinase AvrPphB susceptible1 (PBS1) has long been considered to play an important role in the plant immune response (Kim et al., 2016; Pottinger et al., 2020; Qi et al., 2014). We screened a putative candidate gene, *TraesCS2B01G302800*, with a nonsynonymous SNP. This gene was annotated as encoding the serine/threonine‐protein kinase PBS1. However, the expression of this gene exhibited no significant differences between the two parents or between the two extreme pools. We also compared the coding sequences of *TraesCS2B01G302800* among different plant species. High sequence similarity was observed in two durum wheat (*Triticum durum* Desf.) varieties (‘Svevo’ and ‘Zavitan’) and nine common wheat varieties (‘Arinalrfor’, ‘Jagger’, ‘Julius’, ‘Lancer’, ‘Landmark’, ‘Mace’, ‘Norin61’, ‘Stanley’, and ‘SyMattis’) (Supplemental Figure S1), indicating that the homologs were conservative and might endure strong selection pressure. To further verify the function of *TraesCS2B01G302800*, the mutant libraries developed by UC Davis (Krasileva et al., 2017) and Yantai University (10th National Congress on Wheat Genomics and Molecular Breeding, unpublished data, 2019) can be requested to screen candidate gene‐mutated mutants and the performance of these mutants under powdery mildew stress. Protocols in molecular biology, such as transient transfection (virus‐induced gene silencing) or hereditable transformation (transgenosis and gene editing), can provide solid evidence to uncover the mystery of *TraesCS2B01G302800*.

Map‐based cloning is considered to be an effective technique to identify and isolate target genes. *Pm3b* (Yahiaoui et al., 2004), *Pm4* (Sánchez‐Martín et al., 2016), *Pm21* (Ying et al., 2018), *Pm24* (Lu et al., 2020), and *Pm41* (Li, Dong, Li, et al., 2020) were reported to have been cloned by this strategy. In light of our identification of the resistance gene *PmCH7087* in this study, further backcrossing to develop a backcross population or a near‐isogenic line might be necessary to map *PmCH7087* into a smaller region, which could strengthen the mapping results obtained and isolate the gene. In that case, *PmCH7087* can be widely used to enhance powdery mildew resistance in wheat breeding.

### 4.3 Segregant RNA sequencing combined with KASP in a large F_2:3_ family can accelerate gene mapping for wheat

With development of high‐throughput sequencing technologies, BSR‐Seq has become an effective method for rapid discovery of novel genes and genetic markers linked to target genes (Pearce et al., 2014). Although BSR‐seq has many advantages in the study of target genes, there are still some limitations. For example, the SNPs from transcriptome sequencing data are only from exon sequences, whereas the majority of polymorphisms reside in intergenic regions. A combination analysis of BSR‐Seq and resequencing of the two parents might deal with this deficiency. Furthermore, there were strict requirements for the time and method of sampling in BSR‐Seq. Despite all these, BSR‐seq still provides a large number of effective markers for gene mapping and isolation (Nishijima et al., 2017).

Recently, a protocol combining bulked segregant analysis (pooling DNA) and local QTL mapping via KASP genotyping was developed to rapidly map the *Cf‐10* gene to a 790‐kb region in an F_2_ population (Liu et al., 2019). In the present study, we used BSR‐Seq for primary mapping and KASP markers to further map the powdery mildew gene *PmCH7087* in a large F_2:3_ population. As disease resistance always gives rise to transcription factor responses, BSR can not only the map the function but can also help us screen DEGs and transcription factors in mapping regions, especially for regions with limited combinations. Similarly, *TmBr1*, a gene responsible for stem brittleness, was isolated by the MapRseq approach and was present in recombinant cold spots (Deng et al., 2019). Therefore, we believe that combining BSR‐Seq analysis and KASP in the primary mapping region in a large F_2:3_ population can considerably accelerate the isolation of the genes responsible for stress‐related traits, such as disease resistance and resistance to abiotic stresses (drought stress, temperature stress, or mineral deficiency stress).

## CONFLICT OF INTEREST

The authors declare that there is no conflict of interest.

## AUTHOR CONTRIBUTIONS

Haixian Zhan: conceptualization, funding acquisition, writing—original draft, writing—review and editing. Yingli Wang: methodology. Dan Zhang: data curation. Chenhui Du: data curation, investigation. Xiaojun Zhang: investigation. Xiaoli Liu: data curation, investigation. Guangyuan Wang: writing—review and editing. Shuosheng Zhang: conceptualization

## Supporting information

Supplemental Table S1. Predicted information of 20 SNP‐based KASP markers.

Supplemental Table S2. Predicted enrichment analyses of genes underlying the 9.68‐Mb mapping region.

Supplemental Table S3 Predicted physical location of powdery mildew resistance genes on chromosome 2B.

Supplemental Figure S1. Sequence analysis of *TraesCS2B01G302800* in Chinese Spring and other relatives. The coding sequence data of two durum wheat varieties (Svevo and Zavitan) and nine common wheat varieties (Arinalrfor, Jagger, Julius, Lancer, Landmark, Mace, Norin61, Stanley, and SyMattis) were downloaded from the Ensembl database (ftp://ftp.ensemblgenomes.org/pub/current/plants/fasta, accessed 31 May 2021). The Protein Basic Local Alignment Search Tool (BLASTP, https://blast.ncbi.nlm.nih.gov/Blast.cgi?PROGRAM=blastp&PAGE_TYPE=BlastSearch&LINK_LOC=blasthome, accessed 31 May 2021) was used to align the coding sequences of the data.
